# P-1837. Development and Validation of a Novel Multiplex Immunoassay for the Measurement of the Antibodies against Pneumococcal Vaccine Serotypes

**DOI:** 10.1093/ofid/ofae631.2000

**Published:** 2025-01-29

**Authors:** Yumiko Hayashi, Kentaro Kato, Konosuke Morimoto, Koya Ariyoshi, Bhim G Dhoubhadel

**Affiliations:** School of Tropical Medicine and Global Health, Nagasaki University, Nagasaki, Nagasaki, Japan; Institute of Tropical Medicine, Nagasaki, Nagasaki, Japan; Institute of Tropical Medicine, Nagasaki University, Nagasaki, Nagasaki, Japan; Institute of Tropical Medicine, Nagasaki University, Nagasaki, Nagasaki, Japan; Institute of Tropical Medicine, Nagasaki University, Nagasaki, Nagasaki, Japan

## Abstract

**Background:**

Enzyme-linked immunosorbent assay (ELISA) is a traditional WHO recommended method to measure antibody levels against pneumococcal vaccines. However, ELISA is limited in its capacity to assess all 24 pneumococcal vaccine serotypes simultaneously, which is time-consuming, labor-intensive, and it requires a large amount of serum samples. Therefore, we require a high-throughput assay to assess all vaccine serotypes simultaneously and efficiently to assess pneumococcal vaccine impact at a population level.

**Methods:**

Twenty-four pneumococcal polysaccharide antigens were coupled with 24 distinct colored microspheres by employing the carboxyl method. These conjugated microspheres were exposed to the WHO reference serum in a well of a 96-well plate. The WHO reference serum contained 24 serotype-specific pneumococcal antibodies whose concentrations were known. After washing, a mouse anti-human IgG monoclonal antibody (a secondary antibody tagged with phycoerythrin) interacted with the antigen-antibody complex, and then the final reaction was read on the Bio-Plex 200 System (BioRad). We performed assay validation parameters, such as sensitivity, specificity, linearity, and reproducibility according to the WHO recommendation.

**Results:**

A 4.0 log10 of dynamic range was observed in all standard curves specific for 24 serotypes. Depending on specific serotypes, a lower limit of quantitation (sensitivity) varied from 0.40 ng/mL to 10.05 ng/mL. The assays were highly specific for individual serotypes, except for 6A and 6B, and 19F and 19A (some cross-reactivity was observed, which was known and expected among these serotypes). Specificity was >90% in all serotypes, except serotypes 8 (87%) and 14 (59%). Reproducibility trials showed that the coefficient of variation for all serotypes was < 20% (desired limit), except serotype 19A (29.7%) and 23F (25.4%).

**Conclusion:**

We have developed and validated the novel microsphere-based assay. As this assay enables the simultaneous measurement of 24 vaccine serotype-specific antibodies in a single well, it will reduce workload, the need for the amount of serum, and the overall cost. We expect to use this assay in pneumococcal vaccine immunogenicity studies among an elderly population in Japan and among young children in Nepal.

**Disclosures:**

**Konosuke Morimoto, MD, PhD**, Pfizer: Grant/Research Support **Koya Ariyoshi, MD, PhD**, Pfizer: Grant/Research Support

